# Translation of the Prion Protein mRNA Is Robust in Astrocytes but Does Not Amplify during Reactive Astrocytosis in the Mouse Brain

**DOI:** 10.1371/journal.pone.0095958

**Published:** 2014-04-21

**Authors:** Walker S. Jackson, Clemens Krost, Andrew W. Borkowski, Lech Kaczmarczyk

**Affiliations:** 1 German Center for Neurodegenerative Diseases, Bonn, Germany; 2 Massachusetts Institute of Technology, Whitehead Institute for Biomedical Research, Cambridge, Massachusetts, United States of America; Ruhr University Bochum, Germany

## Abstract

Prion diseases induce neurodegeneration in specific brain areas for undetermined reasons. A thorough understanding of the localization of the disease-causing molecule, the prion protein (PrP), could inform on this issue but previous studies have generated conflicting conclusions. One of the more intriguing disagreements is whether PrP is synthesized by astrocytes. We developed a knock-in reporter mouse line in which the coding sequence of the PrP expressing gene (*Prnp*), was replaced with that for green fluorescent protein (GFP). Native GFP fluorescence intensity varied between and within brain regions. GFP was present in astrocytes but did not increase during reactive gliosis induced by scrapie prion infection. Therefore, reactive gliosis associated with prion diseases does not cause an acceleration of local PrP production. In addition to aiding in *Prnp* gene activity studies, this reporter mouse line will likely prove useful for analysis of chimeric animals produced by stem cell and tissue transplantation experiments.

## Introduction

Prion diseases, formally known as transmissible spongiform encephalopathies, are a group of neurodegenerative diseases most infamous for their ability to spread between individuals [1]. Prion diseases have propagated in sheep and goats for centuries [2]. A more recently emerging prion disease called chronic wasting disease, affecting deer, elk, and moose in the wild, is causing great concern since it is spreading rapidly across North America [3], [4] and might spread to household animals [5]. The apparent transmission of prion disease of cattle to humans that consumed them has exacerbated those concerns [6], [7]. Fortunately, it is exceedingly rare for exogenous prions to infect and trigger disease in humans, historically accounting for less than 1% of all cases of human prion disease [8]. The infectious agent (prion) is thought to encipher the disease inducing information in the conformation of misfolded PrP [9]. During the process of prion replication, a small fraction of the normal non-disease related form (PrP^c^) converts into an aggregated form that is partially resistant to digestion by proteinase K (termed PrP^Sc^ or PrP^res^), which is thought to represent the infectious form [9].

Although rare and infectious, prion diseases share important features with more common neurodegenerative diseases, such as Alzheimer’s (AD) and Parkinson’s diseases. For example, human prion diseases can be caused by familial mutations (∼ 15% of cases) but they are more commonly sporadic (∼ 85% of cases), meaning they arise for undetermined reasons [8]. They generally appear late in life, which is especially intriguing in the familial cases where the mutant gene is expressed harmlessly for many decades [10]. Most neurodegenerative diseases appear to be caused by misfolding of specific proteins, which often aggregate as the disease progresses. Like most neurodegenerative diseases, prion diseases also result in reactive astrocytosis. Despite these similarities, each neurodegenerative disease typically begins in specific brain regions for reasons currently not understood [11]. PrP is especially interesting in this regard as different mutations to this single gene (*Prnp*), which is widely expressed throughout the brain, target different brain regions and cause different clinical signs. Creutzfeldt-Jakob disease typically targets the cortex and causes cognitive problems, Gerstmann-Sträussler-Scheinker syndrome usually targets the cerebellum and causes motor control problems, and fatal familial insomnia most prominently target the thalamus and causes sleep and autonomic dysfunctions. Understanding how this single protein, PrP, can selectively target different brain regions in these different diseases could reveal the general mechanisms of selective vulnerability for other neurodegenerative diseases.

Identifying the specific brain cell types that express PrP will be an important first step to understand the mechanism of selective vulnerability. However, this is problematic for PrP for two reasons. First, PrP is loosely attached to the outer layer of the cell membrane by a glycosylphosphatidylinositol anchor, and therefore resides in a fluid environment, even following tissue fixation [12]. Second, because PrP is targeted to an extracellular location distal from the cell body, it can be difficult to attribute that proportion of protein labeling to a specific cell [13]. These features may have resulted in contradictory reports of the distributions of PrP and *Prnp* mRNA. For example, *Prnp* mRNA was reported to be present in glial cells [12], [14], [15], but the protein is often reported to be absent [12], [16]–[18] though some have detected the protein on astrocytes [19]. These discrepancies beg the question: was it really *Prnp* mRNA and protein or were other RNAs or proteins detected by the probes? Definitive proof could be obtained by manipulating the endogenous *Prnp* gene and then detecting the corresponding change to the gene product. If *Prnp* mRNA is present in glia is it translated or rather maintained in a silenced state? Aggregated PrP has been found in astrocytes in prion diseased brains, but it is unclear whether the astrocytes themselves made the toxic PrP aggregates or were induced to engulf them [20]. PrP made by glia can theoretically contribute to a natural prion disease process as demonstrated by the finding that transgenic mice expressing PrP directed by a glial fibrillary acidic protein (GFAP) promoter are vulnerable to prion infection [21]. Therefore, glia might be an important source of PrP during disease.

Another question is whether the prion protein gene activity is dynamic and adjusts in response to certain conditions [22], which is important for at least two reasons. First, acquired prion diseases typically have a very long preclinical phase, with no signs of disease, followed by a rapid and very short clinical phase, associated with a dramatic increase in the amounts of PrP^res^. Why there is such a rapid transition from a clinically healthy state to a terminally ill state is poorly understood. In mouse models, PrP levels rapidly increase late in disease [23], likely a pathological event, and determining whether this is due to increased synthesis or reduced degradation will yield important clues into the disease mechanism. Second, prion disease and Alzheimer’s disease related aggregates have been found in the same brains [24]–[32]. Moreover, PrP can interact with toxic beta-amyloid oligomers associated with Alzheimer’s disease [33]–[36], which may directly modulate NMDA receptor conductance [37]. A better understanding of *Prnp* expression activity across the brain is warranted.

For these reasons we sought to develop a tool to study *Prnp*’s activity within the context of the vast assortment of neuronal and non-neuronal cell types of a living mammalian brain. Knock-in mice were engineered so that the coding sequence of PrP was replaced with one encoding GFP. This knock-in line was named ki-Prnp-GFP to distinguish it from transgenic mice expressing GFP and GFP-PrP fusions driven by randomly integrated *Prnp* promoter elements. Native fluorescence from unfixed and lightly fixed ki-Prnp-GFP brain slices was easily observed, with intensity differences across and within brain regions, although excessive fixation inactivated the fluorescence. GFP was detected in astrocytes, providing strong evidence that *Prnp* mRNA is present and translated there. However, under conditions of reactive gliosis, GFP did not increase, indicating the dramatic increase in PrP levels during acquired prion diseases is not due to increased synthesis but rather, reduced degradation.

## Results

**Figure 1:**
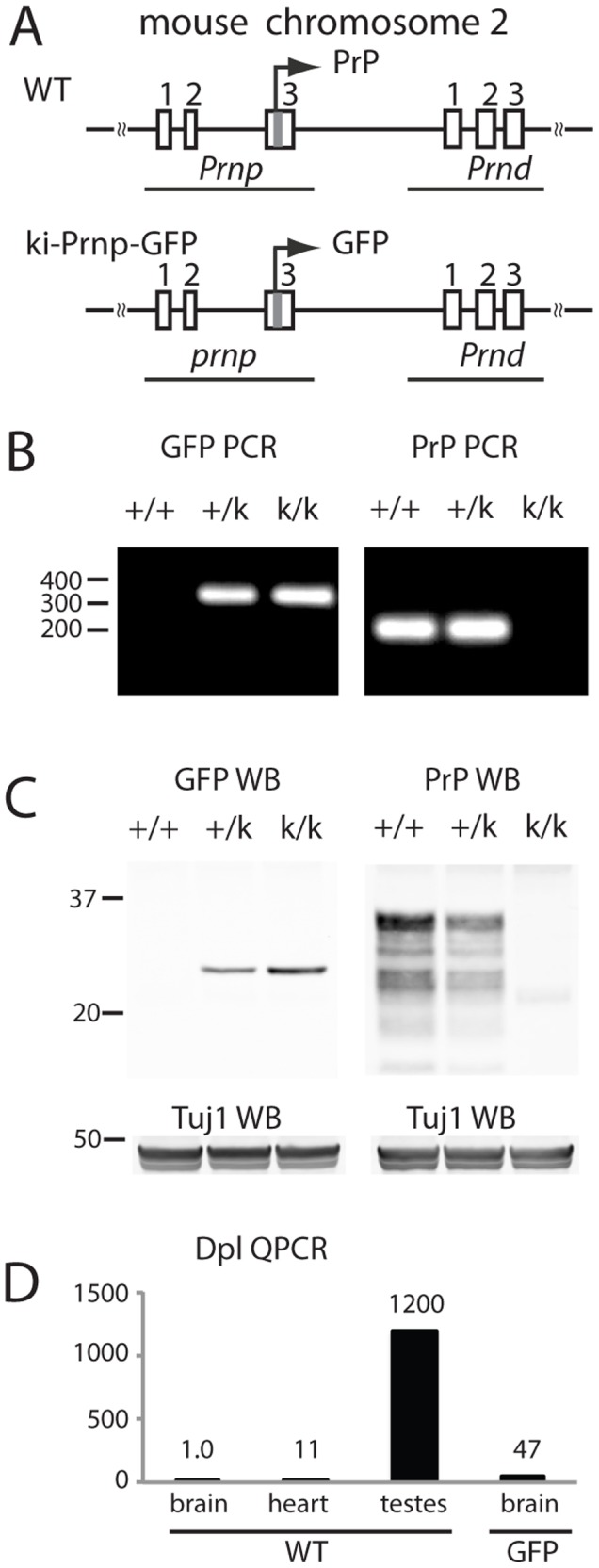
Prnp manipulation. (A) The PrP protein coding sequence was replaced with GFP protein coding sequence. Rectangles represent exons, which are numbered above. The horizontal line connecting the exons represents DNA and the squiggly lines indicate the continuity of the chromosome. The position of genes *Prnp* and *Prnd* are indicated. The arrow above exon 3 indicates the direction and translation start location of *Prnp* (B) PCR of DNA from WT (+/+), heterozygous (+/k) and homozygous (k/k) knock-in mice. GFP specific primers (285 bp expected product) do not amplify DNA from WT mice and PrP specific primers (204 bp expected product) do not amplify DNA from k/k mice. (C) Western blot analysis using antibodies against GFP or PrP (SAF61) show that in +/k mice GFP and PrP levels are reduced to about 50% of homozygous. Tuj1 served as a loading control. (D) QPCR of RNA samples indicate *dpl* mRNA is higher in ki-Prnp-GFP k/k brain than in WT brain, but is still far lower than the amount shown to induce degeneration (∼ equal to levels in testes). The Y axis represents relative expression levels with WT brain set to 1.

To substitute GFP for PrP coding sequence in the *Prnp* gene locus of mouse embryonic stem (ES) cells we employed a gene-targeting procedure similar to the one we used previously [38]. In this design, only exon 3 is modified, leaving all other exons, introns, promoter regions and the 3′ untranslated region intact (Figure 1A). Ki-Prnp-GFP mice were then produced from correctly modified ES cells, identified by PCR and Southern analysis. Matings of heterozygous mice generated progeny with predicted Mendellian ratios and homozygous mice of both sexes were fertile. Genotypes were determined with two separate PCR reactions, one detecting the knock-in GFP allele and the other the unmodified PrP allele (Figure 1B, left and right, respectively). The levels of PrP and GFP in brains correlated with the genotypes of the mice, where heterozygotes had approximately half the levels of homozygotes (Figure 1C). Quantitative real-time PCR (qPCR) of purified mRNA revealed lower levels of ki-Prnp-GFP mRNA (∼ 50 to 60% of WT, data not shown), possibly due to reduced stability of the mRNA or due to sorting problems since the mRNA is normally translated while attached to the ER but is now encoding a cytosolic protein.

*Prnd*, a downstream gene (Figure 1A) encoding the doppel (Dpl) protein, is normally expressed at high levels in testes, and much lower levels in heart and brain [39], [40]. In brains of some Prnp-KO mouse lines its expression is pathologically upregulated to levels exceeding that in testes, apparently due to splicing errors caused by a missing exon 3 splice acceptor site [39], [40]. Since the ki-Prnp-GFP allele retained this splice acceptor site we were surprised to find that *Prnd* message was also aberrantly expressed in ki-Prnp-GFP mouse brains, though it was still much lower than that in testes (Figure 1D). Importantly, heterozygous and homozygous ki-Prnp-GFP mice showed no obvious behavioral or neuroanatomical abnormalities, even in mice aged to 18 months (data not shown). This was partially expected since conventional Prnp-KO mice are normal, but it also indicates that the high expression level of GFP, and the elevated expression of Dpl was not overtly toxic.

### Native Fluorescence of GFP has Regional Differences

We next examined the spatial distribution of GFP in brain slices. Sections were cut from lightly fixed brains and in some experiments sections were treated with a fluorescent Nissl counter stain. Two findings were immediately apparent: the expression of GFP was sufficiently high for direct visualization of its native fluorescence, and the fluorescence intensity varied between brain regions. The same mosaic expression patterns were observed with bright field microscopy experiments using antibodies against GFP (data not shown), verifying that the fluorescence in the green channel was produced by GFP.

**Figure 2:**
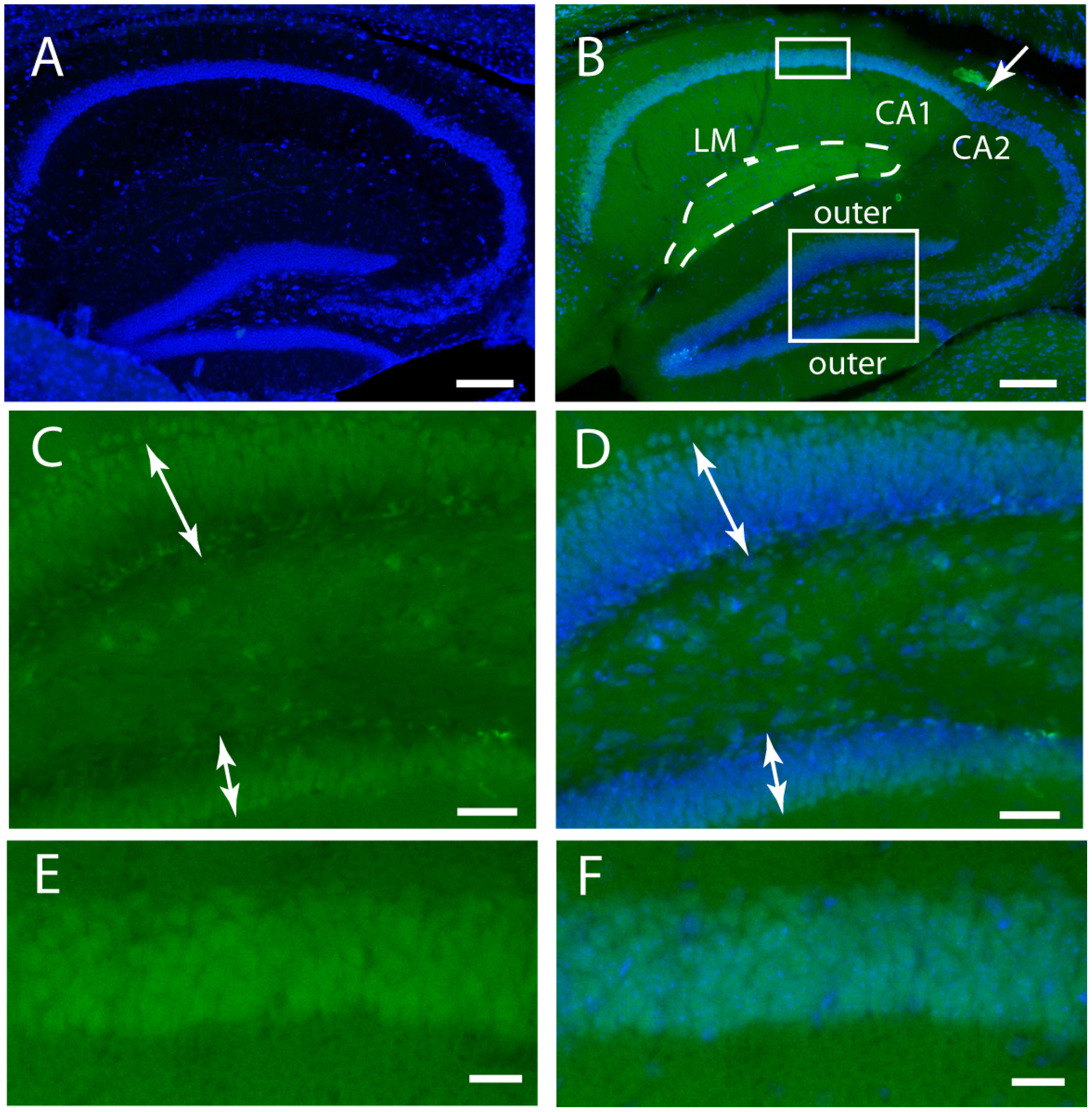
Native GFP fluorescence in hippocampus. (A) WT mice have no detectable fluorescence in the green channel. The blue signal (A, B, D, F) is a Nissl dye used for a neuron specific counterstain. (B) The low magnification of a ki-Prnp-GFP k/k hippocampus shows the CA1 and CA2 boundary indicated by an arrow and LM marked with a dashed loop. The small rectangle on the top marks the region expanded in (E) and (F). The large box at the bottom marks the region expanded in (C) and (D). (C) GFP intensity gradient across the two blades of the dentate gyrus, shown with Nissl counterstain in (D). (E) Heterogeneous fluorescence intensity in the CA1, shown with counterstain in (F) by dark blue dots (indicates cells with no GFP). Scale bars correspond to 200 µM in A and B, 50 µM in C and D, and 25 µM in E and F.

The hippocampus provides an interesting example of the non-uniform distribution of fluorescence. Native GFP fluorescence was undetectable in WT brains (Figure 2A). In contrast, the ki-Prnp-GFP brains generated easy to observe fluorescence (Figure 2B). Interestingly, in the neuropil areas which are rich in synapses but have few neuronal cell bodies, there was a distinct boundary between CA1 and CA2 regions (Figure 2B, arrow) where CA1 was more intensely fluorescent. Moreover, within the CA1, the lacunosum moleculare (LM) has an even higher fluorescence intensity, which is interesting since this layer has the highest density of histopathology (spongiosis and PrP aggregates) in a *Prnp* knock-in mouse model of Creutzfeldt-Jakob disease [41]. There were also fluorescence intensity gradients across the thickness of the cell body layers. For example, in the granular cell layers of the dentate gyrus, outermost regions were more intensely fluorescent than inner regions (Figure 2C, D). A fluorescence gradient was also observed across the thickness of the CA1 pyramidal cell layer with fluorescence intensity higher in the ventral than the dorsal edges (Figure 2E, F). Although these layers appear to be morphologically homogeneous, they have complex expression pattern gradients for many genes [42].

**Figure 3:**
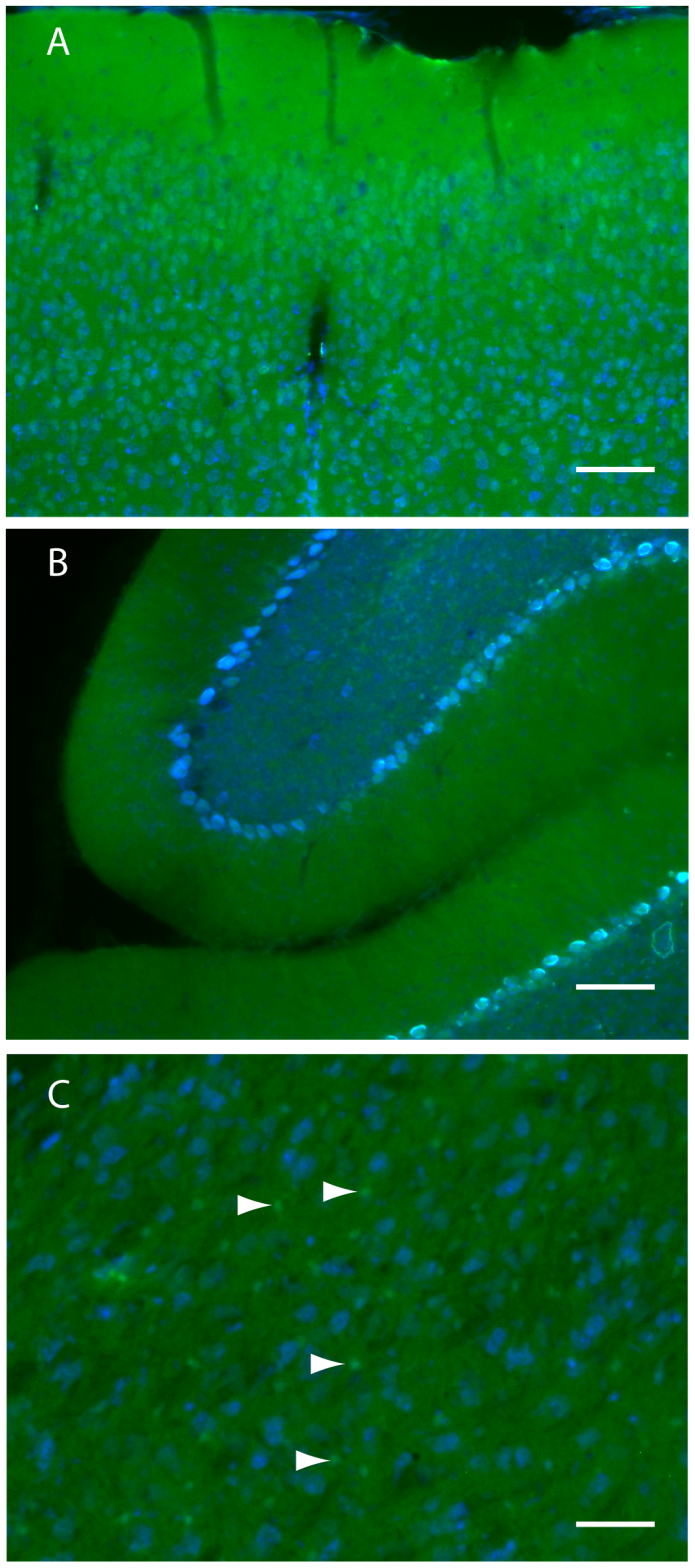
Native GFP fluorescence in other regions of ki-Prnp-GFP k/k brain. (A) Neurons in the cortex have various levels of GFP. Cells with high amounts appear green as it outcompetes the blue signal; cells with moderate amounts appear light blue due to blue/green spectral mixing; cells with low or no GFP appear blue. (B) Cerebellum exhibit diffuse GFP fluorescence throughout the molecular (m) and granular (g) layers. Most Purkinje cells are light blue (moderate GFP levels) but some are dark blue (low GFP levels). (C) The thalamus has many small bright green cells, with high GFP and no blue Nissl (arrowheads). Scale bars represent 100 µM (A and B) or 50 µM (C).

Examination of other brain regions revealed additional complex patterns of GFP expression. For example, the cortex had many cells that were intensely GFP fluorescent, especially in the outer layers, and others that appeared to be negative, (Figure 3A). Many other regions, including the striatum, brainstem, olfactory bulb, also had mosaic GFP fluorescence patterns (data not shown). In contrast, the granular, molecular, and Purkinje cell layers of the cerebellum each had a rather uniform GFP fluorescence intensity (Figure 3B). Interestingly, a small number of Purkinje cells were GFP negative (Figure 3B), an observation reported previously [43]. Importantly, many regions had intensely positive foci that were negative for the Nissl counterstain and approximately the size of glial cell bodies (Figure 3C, arrowheads).

### *Prnp* is Active in Astrocytes

**Figure 4:**
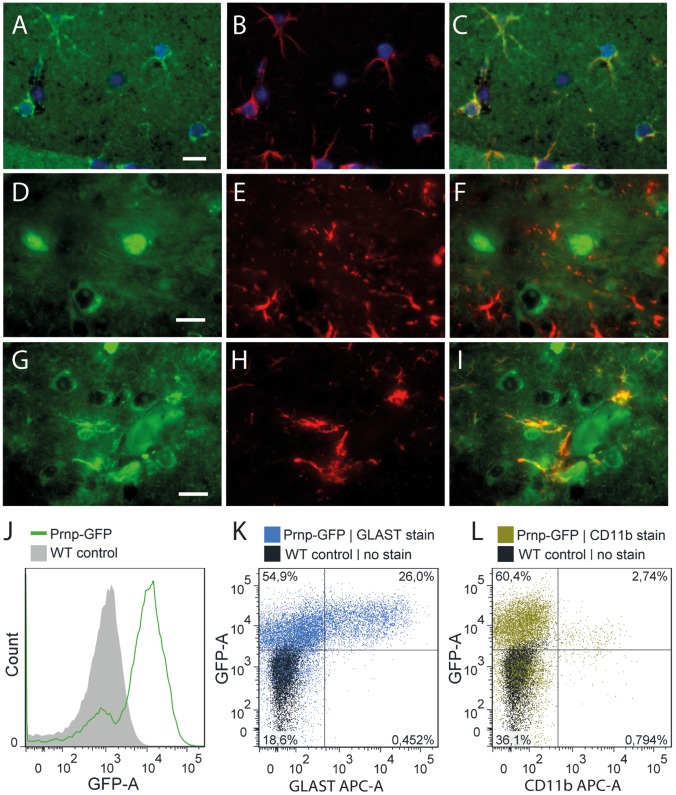
Immunolocalization of PrP, GFP and GFAP in ki-Prnp-GFP +/k brain. (A) In uninfected brains, GFP is observed in astrocyte-shaped cells, which are GFAP+ (B, C). In order to increase the astrocyte size and therefore enhance staining and imaging, brains with middle stage (week 41 of 54 incubation course) prion-induced reactive gliosis were studied (D–I). (D) PrP (green) is mostly diffusely extracellular and intracellular in some neurons, but does not co-localize with GFAP (red) of astrocytes (E, F). Nuclei were counterstained with DAPI. (G) GFP is present in some cell bodies and co-localizes with GFAP (E, F). (J–L) FACS analysis of dissociated ki-Prnp-GFP brains. (J) Histogram distribution of GFP fluorescence in ki-Prnp-GFP k/k mice shows distinct GFP- and GFP+ populations, comprising ∼17% and ∼83% of cells respectively. (K) Most GLAST+ cells are GFP+ and their GFP fluorescence intensity is higher than for GLAST- cells (medians of populations are 0.6×10^4^ for the top left quadrant, and 1.5×10^4^ for the top right quadrant). (L) CD11b+ cells show lower GFP fluorescence intensity then CD11b− cells (medians of populations were 1.1×10^4^ for the top left quadrant, and 0.57×10^4^ for the top right quadrant). Scales in A, D and G represent 10 µM.

To determine if the small GFP fluorescent spots were astrocytes, we performed immuno-colocalization studies of GFP and astrocyte specific marker proteins GFAP and GLAST (glutamate aspartate transporter). Brain sections stained with GFP antibodies revealed a somewhat diffuse staining, with some small astrocyte shaped cells being intensely GFP immunopositive (Figure 4A). Importantly, these GFP positive structures were also positive for GFAP, indicating they had both morphological and molecular characteristics of astrocytes (Figure 4B, C). Similar cells were not positive for Iba1, indicating they were not microglia (data not shown). Even though it is generally detectable in only a subset of normal astrocytes, GFAP is a popular marker for astrocytes since it highlights their star-like morphology [44], [45]. Therefore, to enhance visualization of GFAP+ astrocytes we induced reactive gliosis by infecting GFP/WT heterozygous mice with scrapie prions, and visualized the brains at middle stages of disease (week 41 of a 54 week incubation period). Staining with antibodies against PrP and GFAP revealed very little co-localization (Figure 4D–F). In contrast, GFP was strongly present in GFAP+ cells (Figure 4G–I). The identity of these cell types was confirmed using fluorescent activated cell sorting (FACS) of uninfected brains. Native GFP fluorescence provided the marker for *Prnp* expressing cells, and fluorescently conjugated antibodies against GLAST or CD11b identified astrocytes and microglia, respectively. Most cells in ki-Prnp-GFP brains were natively fluorescent (Figure 4J). Remarkably, nearly all GLAST positive cells were strongly GFP positive and the cells with the highest GFP fluorescence were GLAST+ (Figure 4K). In contrast, CD11b positive cells were only weakly GFP positive (Figure 4L), indicating *Prnp* produces a transcript that is intensely translated in astrocytes but only weakly translated, if at all, in microglia.

Reactive gliosis is a common feature of many neurodegenerative diseases, identified as an increase in size, and sometimes number, of astrocytes [46]. We therefore wondered if in the course of reactive gliosis, *Prnp* gene activity would also increase. Prion disease was induced in heterozygous ki-Prnp-GFP/WT mice by intracranial injection of a common laboratory strain of mouse adapted goat prions (RML). Mice were then sacrificed at various time points following the injection, and their brains were analyzed for changes in PrP and GFP levels. The incubation period to terminal disease was 12.5 months, as expected since prion disease progresses slower in heterozygous mice than in WT mice due to the reduced levels of PrP present [47], [48].

**Figure 5:**
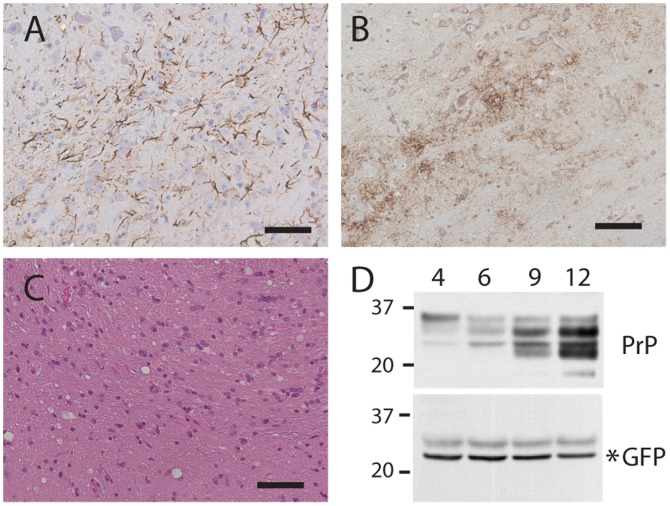
RML scrapie infection induces accumulation of PrP but not GFP in heterozygous ki-Prnp-GFP/WT mice. Reactive gliosis (A), PrP^res^ (B), and spongiosis (C) are apparent in brains following a 41 week (9 months) incubation. Brains harvested at 4, 6, 9, and 12 months after infection show increasing PrP (D, top blot), but not GFP (D, lower blot). Molecular weight markers are on the left, and time post infection (months) is indicated above the blots. Scale bars in A–C represent 50 µM.

Beginning at six months of infection and progressing steadily throughout the disease process, classic features of mouse scrapie histopathology were observed, including reactive gliosis, PrP^res^, and spongiform degeneration (the classic sponge-like appearance that is a hallmark of prion diseases (Figure 5 A–C)). Total levels of PrP (PrP^res^ plus PrP^c^) in brain homogenates also increased with disease progression, indicating that in addition to converting to PrP^res^, PrP is accumulating (Figure 5D). However, GFP did not increase as disease progressed (Figure 5). Comparison of immunofluorescence staining of brains that were uninfected or at various stages of scrapie infection did not reveal any obvious increases in GFP levels, other than some GFP+ astrocytes appearing to be larger (data not shown). These observations indicate that the increased levels of PrP occurring during prion infections are not due to increased synthesis, but likely result from impaired degradation.

## Discussion

Several other mouse lines expressing GFP reporters have been generated [16], [49]–[51]. For example, many lines express PrP::GFP fusion proteins which apparently traffic though the secretory pathway as well as WT PrP does [16], providing a terrific resource for imaging studies of PrP trafficking in living cells [52]. Such constructs can label PrP aggregates induced by prion infections [51], [53], paving the way for advanced *in vivo* imaging experiments, potentially investigating how aggregates move across the brain [11]. However, these models may not accurately report *Prnp*’s normal expression pattern throughout the brain. One reason for this caveat is that these models employed randomly integrated transgenes, which are highly prone to genome position effects. For example, the *Prnp* half-genomic promoter or MoPrP.XhoI, lacking an important intron [54] was shown to be active in the striatum of one transgenic mouse line but not a second [54]. Moreover, PrP is normally expressed in Purkinje cells [12] and sometimes this promoter fragment is active in Purkinje cells [55], but sometimes it is not [43], [54], including in one of the GFP random integration transgenic mouse lines [16]. Since Purkinje cells are among the easiest cell types to identify, this comparison was straightforward, but expression differences in other cell types would be much harder to identify. One mouse line used a complete *Prnp* promoter element [51], but even these types of constructs can show integration effects [56].

Of course, our model also has limitations. One drawback of our ki-Prnp-GFP line is that it gives no information on intracellular PrP trafficking. Moreover, GFP is a cytosolic protein while PrP is a membrane bound, extracellular protein. The two proteins likely have very different interactions with cell-type specific protein quality control machinery, potentially resulting in GFP levels that do not directly correlate with PrP synthesis. Also, our model appears to induce a low level of Dpl expression, which is unlikely true with the other models. However, the increased Dpl expression in homozygous mice was only 4% of that found in testes, the level that induced cerebellar degeneration [39], [40]. Therefore, Dpl expression is likely too low to cause degeneration in ki-Prnp-GFP mice, and since the expression of WT PrP can dominantly suppress Dpl toxicity [39], [40], heterozygous mice expressing both a WT and GFP allele will be even less likely to develop Dpl-induced neurodegeneration.

Despite the potential weaknesses, the unique design of our ki-Prnp-GFP line will nicely complement the previous transgenic lines in a number of ways, as evidenced by the new knowledge of *Prnp* gene activity we uncovered during this initial assessment. For example, unlike for the random integration models [16], [50] our model clearly shows that *Prnp* mRNA is translated in astrocytes. Interestingly, however, like most other groups, we did not detect the protein on astrocytes [12], [16]–[18]. There are several possible explanations for this apparent contradiction. First, glial PrP might simply be hard to detect. PrP is loosely attached to the outside layer of the cell membrane and glial membranes may be more prone to release PrP than neuronal membranes during processing or staining procedures [12]. Second, PrP might be more efficiently processed in astrocytes and trafficked to the cell surface, leaving little intracellular PrP to be detected. Third, PrP might be secreted from glia, and therefore not present in astrocytes long enough for detection. This interesting possibility suggests PrP might serve different roles in different cell types and it might normally function as a signaling molecule for glia, and potentially contribute to neurodegeneration during gliosis. Studies of cells in culture also indicate PrP has important roles in the physiological function of astrocytes [57], [58] with an unexpectedly high level of PrP in the astrocyte preparations compared to the neuron preparations [57]. That these cells, compared to astrocytes in brain, have easily detectable PrP is difficult to explain but may indicate that astrocytes in culture retain more PrP than those in brain, and may also indicate that conventional cultures of astrocytes are quite different from those in mature brain [59]. While cell type specific protein quality control systems may process PrP differently [11], our results indicate that the differences in PrP levels across different brain regions is at least partially influenced by *Prnp*’s differential activity in different cells, consistent with mRNA localization studies [12].

Despite induction of reactive gliosis, scrapie infection did not induce increased levels of GFP. This lack of induction might be because reactive gliosis is often related to an increase in size of astrocytes, with only a small increase in cell number [46], which might also be true for rodent scrapie [60]. In contrast, activation of reporter activity in these mice has been demonstrated by inducing an inflammatory response [61]. Since other biologically interesting challenges appear to involve *Prnp* expression (e.g. ischemia, hypoxia and cancer; [62]–[67]) this mouse line could be a useful resource for researchers interested in studying the dynamics of *Prnp* activity *in vivo*. Moreover, induced pluripotent stem cells from these mice, and the ES cells used to generate them, may be useful for testing stem cell differentiation procedures and therapeutic transplantations for mouse models of many diseases, possibly being observed in living brains through cranial windows. FACS experiments of ki-Prnp-GFP tissues may also be a powerful new tool to study the rare cells that express *Prnp* in tissues where the gene is mostly inactive. Indeed, in our preliminary attempts we isolated a large number of GFP positive cells from blood, heart, intestine, liver, and lungs, a small number from spleen, and none from other tissues, most notably muscle. Clonal cell lines from these mice can be used as a screening platform for agents that suppress or activate *Prnp* activity. The mice will also be quite useful for more thorough investigations of spatial *Prnp* activity through development and in aging.

## Methods

### Ethics Statement

Experiments with live animals were conducted at the Whitehead Institute. Protocol 0702-022-05 “Investigation of the Pathogenesis of Transmissible Spongiform Encephalopathies” was approved by the Division of Comparative Medicine Committee on Animal Care at Massachusetts Institute of Technology and was followed with utmost care to prevent or reduce pain and stress to the animals.

### Genome Manipulation

To generate ki-Prnp-GFP mice we used the same ES gene targeting screening strategy reported [38] with a few changes. Sequence encoding enhanced GFP was obtained from the Clontech vector pBI-EGFP by digestion with XbaI and blunting with Klenow and dNTP, and gel purified. The targeting homology pWJPrP38 was digested with ClaI and EagI, blunted with Klenow and dNTP, dephosphorylated with calf intestinal phosphatase (all enzymes from New England Biolabs), gel purified, then ligated with the GFP fragment, to generate the final targeting vector pWJPrP49. Gene-targeting was done as described [38], using 129/Ola strain HM-1 ES cells [68]. Correctly targeted ES cells were identified by PCR and Southern analysis. Chimeras and their offspring were bred to C57Bl/6N for a total of 4 generations for experiments reported here, and the line was further backcrossed for a total of 9 generations. This line is currently maintained on a congenic C57Bl/6N background and can serve as both a conventional Prnp-KO line and as an indicator line for *Prnp* activity.

Genotyping was performed using PCR primers #75 and #77 yielding a 204 bp band for WT, and #77 and #79 yielding a 285 bp band for Prnp-GFP. Primer sequences: #75 5′-GAGCAGATGTGCGTCACCCAG; #77 5′- GAGCTACAGGTGGATAACCCC; #79 5′-AGATCCGCCACAACATCGAGG; cycling conditions: 95°C 30 sec, 62°C 30 sec, 72°C 1 min, for 30 cycles.

### Western Analysis

For western analysis, 10% brain homogenates were prepared and cleared of nuclei as described [69]. The blotting procedure used precast 10% gels with MES buffer (Invitrogen). For figure 1, antibodies were mouse anti-PrP SAF61 1:2000 (Cayman Chemical), mouse anti-GFP 1∶2000 (Roche), Rabbit anti-Tuj1 1:4,000 (Covance), followed by appropriate fluorescent secondary antibodies and scanned on the Odyssey by Li-cor. The blots in figure 5 were similar except mouse anti-PrP SAF83 (1∶2,000) was used.

### qPCR Analyses

For the analysis of *Prnd* expression, two C57BL/6N WT and two C57BL/6N ki-Prnp-GFP mice (one female and one male each) at seven weeks of age were used. After killing the mice with CO_2_, brains (males and females) and testis (males) were immediately dissected and subsequently used for total RNA isolation. Therefore, all tissue samples were weighed and homogenized with a dounce homogenizer in 10 volumes of hypotonic lysis buffer (50 mM Tris, pH 7.5, 100 mM KCl, 12 mM MgCl2, 1% Nonidet P-40, 1 mM DTT, 1x protease inhibitors and 2,5 µl/ml RNAse inhibitors. Homogenates were centrifuged for 10 min at 10000 g and 4°C. 200 µl of the supernatant was used for total RNA was isolated using the RNeasy Mini Kit (Qiagen, Hilden, Germany) including on-column DNAse digestion. Integrity and quantity of all RNAs was determined using a 2100 Bioanalyzer (Agilent, Santa Clara, USA) and a Qubit 2.0 Fluorometer (Invitrogen, Carlsbad, USA). For each reverse transcription (RT) reaction (using TaqMan Reverse Transcription Reagents, Life Technologies, Carlsbad, USA), 500 ng of high quality RNA (RIN >8.5) was used. Additionally, 50 ng of apple (*Malus x domestica*) leaf RNA was spiked into each reaction as an RT efficiency control. Each qPCR was performed in triplicates using Power SYBR Green PCR Master Mix and a 7900HT Fast Real-Time PCR System (both Applied Biosystems, Carlsbad, USA). Amplicon sizes were chosen to be size-matched for *Prnd* (114 nt) and *Prnp* (113 nt) and both derived from the 3′UTR of each gene using the following primers for *Prnd* (for 5′-CAGGGCGGTGGATACCTT-3′; rev 5′-GGCAGAGGGAGGAGATGG-3′) and *Prnp* (for 5′-ACATCTGAAGTATGGGACGC-3′; rev 5′-TAGGGGTCTGCTTTGGAATC-3′). All Ct values were normalized to the spiked control (using a *Malus x domestica* ADF assay) and relative expression levels were determined using ΔCt method.

### Native Fluorescence

To preserve native fluorescence, brain hemispheres were immersion fixed in 2.5% formalin for 4 hours, then cut on a vibratome into 50 micron thick sections. The sections shown in figures 2 and 3 were also stained with fluorescent counter stains Nissl (1∶300 in PBS) and myelin (1; 200, though presenting only Nissl). Imaging parameters were set so that WT brains gave no signal in the green channel.

### Immunofluorescence

Brains were fixed by immersion in formalin. Scrapie infected samples were subsequently treated with 96% formic acid for 1 hour, rinsed in PBS, then placed into fresh formalin overnight. Tissues were embedded in paraffin and 4 micron thick sections were cut. Following standard dewaxing steps, epitope retrieval was performed by heating sections to a boil in 10 mM citrate buffer pH 8.0 and allowed to cool for 30 min, followed by a 3 minute soak in 98% formic acid. Sections were then neutralized and labeled with anti-GFAP 1∶3,000 (Mab360 Chemicon) using a MOM kit from Vector (FMK-2201), subsequently blocked with a biotin blocking kit (Vector SP-2001) then stained with either mouse anti-PrP SAF32 1:200 (Cayman Chemical) or with mouse anti-GFP 1∶200 (Roche, 11814460001) using the MOM kit with a Texas-red avidin. Autofluorescence eliminator (Millipore) was used prior to cover slipping. To ensure there was not cross labeling with second secondary kit against the first primary, in some experiments we followed the PrP or GFP labeling with labeling with Alexafluor 555-conjugated mouse anti GFAP (Cell Signaling, 3656). We also performed reverse experiments where the colors were reversed and or the order of primaries used was reversed, with the same result.

### FACS

Mice (Prnp-GFP k/k and age-matched WT) were sacrificed at postnatal day 5. Brains were chopped in ice-cold HBSS and dissociated with trypsin-based Brain Tissue Dissociation Kit (Miltenyi Biotec), following the manufacturer’s protocol with minor modifications. Cell suspensions were diluted to ∼5×10^6^ cells/ml, and aliquots thereof stained with APC-coupled anti-CD11b and anti-GLAST antibodies (Miltenyi Biotec). Prior to FACS, suspensions were filtered through 70 uM mesh and stained with PI to discriminate dead cells. Data were acquired using FACS Canto II flow cytometer (BD Biosciences) and analyzed with FlowJo 7.6 (Tri Star, Inc.).

### Scrapie Infections

The RML strain of mouse adapted scrapie prions were injected intracranially into isoflurane anesthetized mice at a dose of 10**^5.5^** infectious units in a volume of 30 µl. Inocula were administered with a 1 ml disposable syringe capped with a 25 gauge intradermal needle and a guide that controls the injection depth to ∼ 3.5 mm, through the junction of the frontal and parietal skull plates, midway between Bregma and the eye socket. The injection was done on a single cohort at the same time. Mice were sacrificed at 4, 6, 9, and 12 months after infection. Immediately following sacrifice, brains were removed, rinsed in cold PBS to remove excess blood, cut down the mid-line, and half frozen on powdered dry ice and the other half emersion fixed in 10% buffered formalin. The cohort included, for each time point, two ki-Prnp-GFP mice, heterozygous for wild-type and GFP alleles.
